# Lung cancer incidence attributable to residential radon exposure in Finland

**DOI:** 10.1007/s00411-022-01004-1

**Published:** 2022-11-08

**Authors:** Olli Kurkela, Jaakko Nevalainen, Salla-Maaria Pätsi, Katja Kojo, Olli Holmgren, Anssi Auvinen

**Affiliations:** 1 STUK—Radiation and Nuclear Safety Authority, Environmental Surveillance, Helsinki, Finland; 2 Faculty of Social Sciences, Tampere University, Unit of Health Sciences, P.O. Box 100, 33014 Tampere, Finland; 3 Laurea University of Applied Sciences, Ratatie 22, 01300 Vantaa, Finland

**Keywords:** Radon exposure, Lung neoplasms, Smoking, Population health, Risk evaluation and mitigation, Environmental exposure

## Abstract

This study aimed to estimate (1) the number of avoidable lung cancer cases attributable to residential radon in Finland in 2017, separately by age, sex, dwelling type and smoking status, (2) the impact of residential radon alone and the joint effect of residential radon and smoking on the number of lung cancers and (3) the potential decrease in the number of radon-attributable lung cancers if radon concentrations exceeding specified action levels (100, 200 and 300 Bq m^−3)^ would have been mitigated to those levels. Population-based surveys of radon concentrations and smoking patterns were used. Observed radon levels were contrasted with 25 Bq m^−3^ representing a realistic minimum level of exposure. Lung cancer risk estimates for radon and smoking were derived from literature. Lastly, the uncertainty due to the estimation of exposure and risk was quantified using a computationally derived uncertainty interval. At least 3% and at most 8% of all lung cancers were estimated as being attributable to residential radon. For small cell carcinoma, the proportion of cases attributable to radon was 8–13%. Among smokers, the majority of the radon-related cases were attributable to the joint effect of radon and smoking. Reduction of radon exposure to 100 Bq m^−3^ action level would eliminate approximately 30% of radon-attributable cases. Estimates were low compared with the literature, given the (relatively high) radon levels in Finland. This was mainly due to the lower radon levels and higher smoking prevalence in flats than in houses and a more realistic point of comparison, factors which have been ignored in previous studies. The results can guide actions in radon protection and in prevention of lung cancers.

**Supplementary Information:**

The online version contains supplementary material available at 10.1007/s00411-022-01004-1.

## Introduction

Radon-222 is a naturally occurring alpha-emitting radionuclide that is generated in the decay chain of ^238^U. In this paper, the term ‘radon’ refers to both ^222^Rn and its progeny that cause a substantial proportion of radiation exposure. Radon causes residential exposure, because ^222^Rn transports easily in soil and can enter buildings, provided that the soil is permeable and entry to a building is possible through cracks or gaps (e.g., unsealed junctions or inlets) in the foundation. Radon concentration in buildings depends on several factors, including soil composition (uranium concentration, permeability) and building characteristics (construction of the foundation, type of basement, number of floors, ventilation affecting passage of radon out of the building). These features are difficult to assess and often result in large variation even between adjacent buildings. Also, climate and weather affect residential radon, as buildings have a negative pressure due to indoor versus outdoor temperature differential in cold conditions. The highest radon concentrations occur in houses, particularly in spaces facing the ground. In flats, radon concentrations tend to be low above the ground floor. Building materials, untreated groundwater and life style factors (ventilation, opening of windows and doors, etc.,) can also contribute meaningfully to indoor radon and in flats, building materials are also commonly an important source of radon.

Inhaled radon (more specifically, its short-lived progeny) causes radiation dose to the epithelial cells of the airways. It is a major contributor to natural radiation worldwide, and the main source of radiation exposure overall in many countries, with average annual effective doses up to 1–5 mSv (Radiation and on the E of A 2008a). It is noted that ionising radiation exposure from medical applications has increased during the past decades. Currently, computerised tomography contributes more than half of all medical radiation exposure in most high developmental index countries (UNSCEAR 2022). The total effective dose for medical exposure, however, is estimated to be < 1 mSv per year globally and 1.7 mSv per year in the highest income level populations (ibid), while the global average effective dose from radon is estimated to be 1.15 mSv per year and as high as 1.6 mSv per year in Finland with substantial international variation. (Radiation and on the E of A 2022).

Radon is an established human carcinogen (International Agency for Research on Cancer, 1988), the second most important cause of lung cancer after tobacco and in many countries, and the main cause among never-smokers (United States Environmental Protection Agency; World Health Organization). An increased risk of lung cancer was first shown among uranium miners occupationally exposed to exceedingly high concentrations, up to > 10,000 Bq m^−3^ (Lubin et al. 1995). A modestly increased risk has subsequently also been demonstrated for residential radon exposure (Darby et al. 2005; Krewski et al. 2005). As for other types of radiation, the risk appears to increase in a linear fashion with exposure without a threshold. Of the lung cancer types, the highest risk coefficient has been shown for small cell carcinoma (Darby et al. 2005). The evidence concerning possible excess risk of other cancer types is inconclusive.

Finland has exceptionally high average residential radon concentrations owing to its geographic, climate and building features. The national arithmetic mean activity concentration in houses has been estimated as 120–145 Bq m^−3^ and in flats 49–82 Bq m^−3^ based on two nationwide measurement surveys (Arvela et al. 1993; Kinnunen et al. 2009). Comparable average national concentrations for residential radon have been reported from Sweden, Estonia and Austria, while a clearly higher national average has been documented only for the Czech Republic. Internationally, the mean national levels range commonly from 40 to 80 Bq m^−3^ (Radiation and on the E of A, 2008b).

In this study, the aim was to estimate the number of lung cancers attributable to radon in Finland in 2017. To achieve this, radon measurements from two national surveys with comprehensive lung cancer incidence data were used. Estimates of lung cancer risk due to residential radon were obtained from pooled epidemiological studies. The analysis was performed by age, sex, smoking history and, due to the inverse correlation between smoking prevalence and radon exposure, by dwelling type. A separate assessment was performed for small cell carcinoma. A distinction between lung cancers attributable to radon alone and those resulting from the joint effect of radon and smoking was made. In addition, the potential impact of radon mitigation in high-radon dwellings to established action levels was assessed. Lastly, uncertainty in the estimates was assessed using a computational approach accounting for the uncertainty in all the key factors: the risk coefficients, the smoking exposure and the radon concentrations. Information provided by the study can be utilised in radiation protection. Because national estimates do not easily transfer to other countries, country-specific studies are of importance (Catelinois et al. 2006; Gray et al. 2009; Ruano-Ravina et al. 2021).

## Materials and methods

### Data sources

**Fig. 1:**
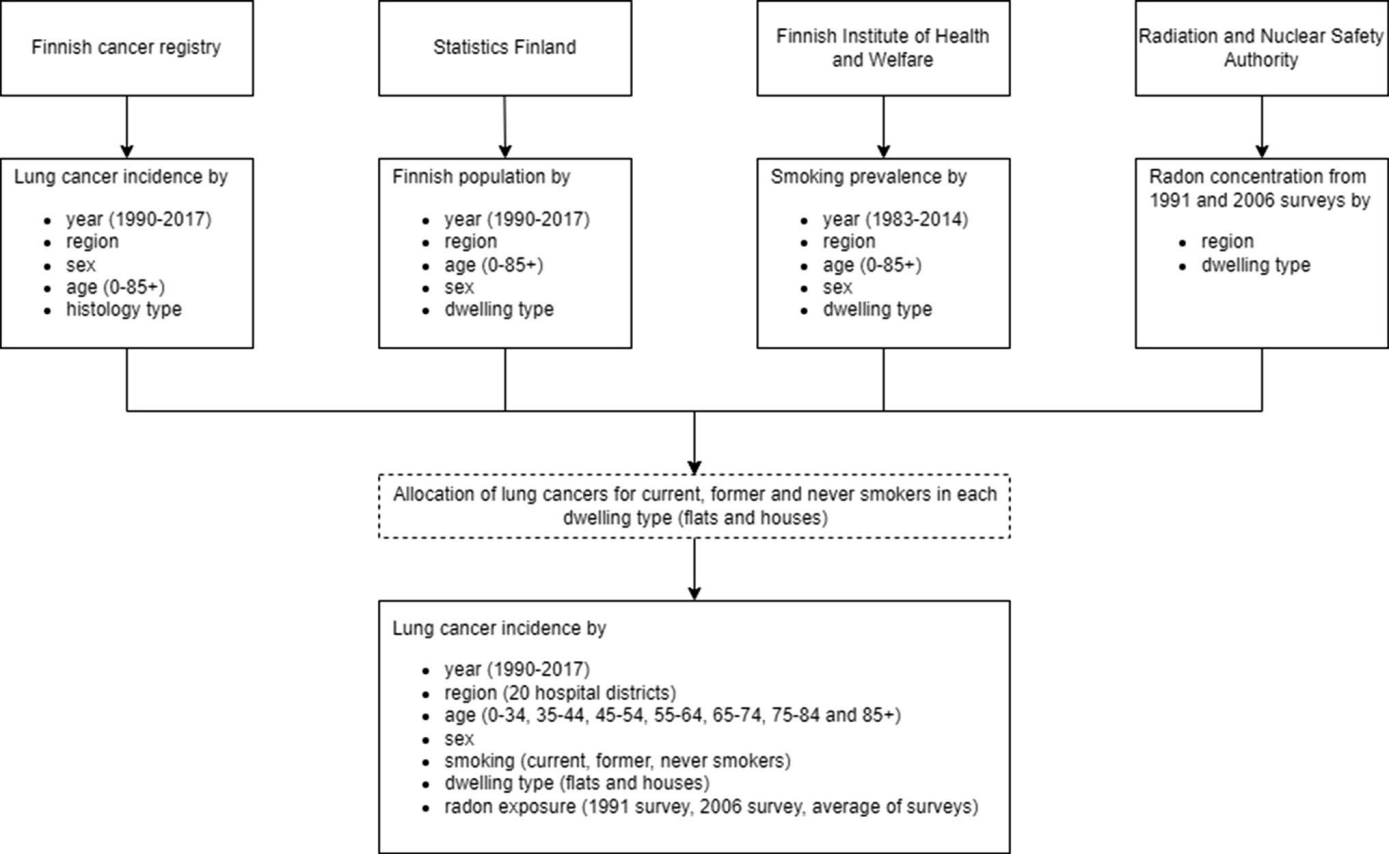
Data sources and allocation of lung cancer cases to each stratum. Year-, age- and sex, and building type-specific smoking prevalence was obtained from the School Health Promotion Study, Health Behaviour and Health among the Finnish Adult Population survey, and Health Behaviour and Health among the Finnish Elderly survey (Helldán and Helakorpi 2012, 2015; Luopa et al. 2014)

Data were compiled from multiple reliable sources (Fig. 1). The numbers of incident lung cancers and the size of population in 2017 were stratified according to region (20 hospital districts), age (0–34, 35–44, 45–54, 55–64, 65–74, 75–84 and 85 + years) and sex.

### Lung cancer incidence

Annual lung cancer incidence data (ICD-10: C33 malignant neoplasm of trachea and C34 malignant neoplasm of bronchus and lung) were obtained from the Finnish Cancer registry, which is a nationwide, population-based cancer registry with a practically complete coverage of lung cancer cases in Finland (Leinonen et al. 2017). The lung cancers were stratified by histologic type (all cancer types and small cell carcinomas). The number of lung cancers from the latest year available at the time of the study (2017) was used.

### Dwelling types

Annual population-level data on dwelling types were obtained from Statistics Finland (Statistics Finland). Dwellings were classified either as houses (low-rise residential buildings including detached and terraced or semi-detached houses) or flats (blocks of flats or multi-story buildings). Often higher radon concentrations are observed on the ground level of the building due to the proximity of the soil, while the radiation on higher levels of the building mainly originates from the construction materials. Typically, the ground level in a flat is uninhabited in Finland. The proportion of people by dwelling type in each stratum was calculated by dividing the number of residents of the dwelling type by the number of people in the stratum.

### Smoking prevalence

Data on smoking prevalence were combined from four national surveys conducted by the Finnish Institute for Health and Welfare (THL). Targeting various age groups, lifestyle-related information including smoking by year, region, age, sex and dwelling type on Finnish adolescent (age 0–19), adult (age 20–64) and senior (age 65 or older) population was regularly collected between 1983 and 2014 (Helldán and Helakorpi 2012, 2015; Luopa et al. 2014).

The surveys did not include smoking prevalence data for the oldest age group (age 85 + years). Therefore, smoking prevalence in this group was estimated using regression. A total of six models were fitted based on the available smoking data to estimate the proportion of current smokers, former smokers and never smokers in the missing age strata.

Furthermore, the surveys lacked information on the dwelling type-specific smoking among adolescents and seniors. Therefore, for population of age 65 or older, similar smoking prevalence by dwelling type to that of population of age 55–64 years was assumed.

For adolescent population, smoking prevalence by dwelling type similar to those of age 45–54 was used, as the adolescents were assumed to still live with their parents.

In the calculations, with the exception of uncertainty estimation, smoking prevalence from the year 2007 was used. The prevalence with a 10-year lag was taken to represent smoking exposure during the time period relevant for lung cancer incidence in 2017.

### Residential radon concentrations

Data on radon concentrations were based on two representative surveys carried out in 1991 and 2006 by STUK Radiation and Nuclear Safety Authority. These were taken to represent population exposure levels for a period of 25 years (excluding a five-year minimum latency, i.e. 1992–2012) prior to lung cancer occurrence (incidence in 2017). Data consisted of 5956 measurements (1991: *n* = 3074, 2006: *n* = 2882) of indoor radon concentration in Finnish dwellings measured with passive alpha track detectors in two consecutive 6-month measurement periods. In case only one measurement was available, it was corrected for seasonal variation (Arvela et al. 1993; Kinnunen et al. 2009). Geometric mean radon concentrations (Bq m^−3^) by region and dwelling type were calculated based on each survey. Residential radon exposure estimates were conditional on dwelling type and region, but within these strata assumed independent of age, sex and smoking.

Analyses were conducted using three sets of estimates for annual regional and dwelling type-specific radon concentrations: (1) radon concentrations based on the first survey in 1991, (2) radon concentrations based on the 2006 survey and (3) the average of the radon concentrations from both surveys. In the analyses, the radon exposure was assumed to have remained constant for the last 5–25 years and was adequately represented by these radon concentrations.

### Lung cancer relative risk due to residential radon

The relationship between lung cancer risk and radon concentration was assumed to follow the model presented in the European pooled study. The pooled European analysis is the largest study on the topic with the most precise risk estimates. In addition, it employed both uncorrected risk estimates and those adjusting for measurement error (corrected risk estimates) (Darby et al. 2006). The linear odds model implies an approximately linear relationship between the relative risk and radon concentration RR ≈ 1 + βX where β stands for excess relative risk (ERR) per 100 Bq m^−3^ increase in radon concentration (X). Using the model, the relative risk was calculated separately for all lung cancers and small cell carcinomas (the subtype with the highest radon-related risk coefficient).

Three estimates of ERR (Darby et al. 2006) were used. First, the uncorrected estimate of an increase of 8.4% (95% confidence interval (CI) 3–16%) in the lung cancer risk per 100 Bq m^−3^ increase in residential radon concentration was used. Second, the corrected estimate from the same study was used, which accounted for uncertainties related mainly to temporal variation (measurement error) in the assessment of residential radon (Lagarde et al. 1997; Heid et al. 2006). This estimate was 16% (95% CI 5–31%) increase in lung cancer risk per 100 Bq m^−3^ increase in radon concentration. For small cell carcinomas, the uncorrected estimate of 31% (95% CI 13–61%) per 100 Bq m^−3^ increase in radon concentration from the European pooled study was used. Despite more recent estimates exist (Rodríguez-Martínez et al. 2022), this estimate is based on the highest number of (nearly 1400 cases) of small cell carcinoma (Darby et al. 2006). Since the European pooled study reported no differences in relative risk by smoking, age or sex, the same ERR estimates for each smoking, age and sex strata were used.

### Lung cancers by smoking category and dwelling type

In each region, age, sex and building type-specific stratum, the lung cancers *(L*_*s*_*)* reported in Finland in 2017 were further allocated for current, former and never smokers based on relative lung cancer risk estimates derived from the literature. A *RR* of 4 for former smokers and 15 for current smokers were used, relative to never smokers (Freedman et al. 2008; Hansen et al. 2018; O’Keeffe et al. 2018). The allocation was then done using the equation *L*_*i*_ = *P*_*ω(i)*_*L*_*s*_*/Σ*_*i*_* P*_*ω(i)*_, where ${L}_{i}$ represents number of lung cancers in each smoking class. Relative risk weighted proportions ${P}_{\omega (i)}$ were obtained as the product of the prevalence in smoking category *i* in 2007 and the literature-based estimate of the relative risk of lung cancer in category *i* compared to non-smokers. Similar relative risks were assumed for both men and women, in accordance with literature (Freedman et al. 2008; O’Keeffe et al. 2018).

### Avoidable radon-attributable lung cancers

The number of radon-attributable lung cancers in Finland in 2017 was assessed in each stratum using the three ERR estimates described above. A similar analysis was carried out for small cell carcinomas.

The number of radon-attributable lung cancers were assessed in each stratum by subtracting the number of lung cancers not attributable to radon from the total number of lung cancers.

These were calculated by dividing the total number of lung cancers in each stratum by the relative lung cancer risk due to radon, calculated using stratum-specific radon concentration and the European radon risk model.

Radon exposure cannot be entirely avoided as radon is present even outdoors. Thus, the number of avoidable radon-attributable lung cancers was estimated by subtracting the number of radon-attributable lung cancers that would have occurred assuming a universal radon concentration of 25 Bq m^−3^ from the number of radon-attributable lung cancers estimated based on the observed residential radon levels. The assumed universal level is approximately equal to the lowest decile of residential concentrations in Finland (Arvela et al. 1993; Kinnunen et al. 2009).

The total number of avoidable radon-attributable lung cancers in the whole population was obtained as the sum across all strata. The population attributable fraction of avoidable lung cancers due to residential radon was calculated as the proportion of avoidable radon-attributable lung cancers out of all lung cancer cases.

### Impact of residential radon mitigation on the number of lung cancers

The potential decrease in the number of radon-attributable lung cancers was estimated in a hypothetical setting, where residential radon concentrations exceeding specified action levels would have been mitigated to those levels. The first action level was based on guidelines by the World Health Organization, which recommends a national annual average radon concentration of 100 Bq m^−3^ (Ruano-Ravina et al. 2017). Second, the action level based on the guidelines by STUK was used, recommending that the indoor radon levels should not exceed 200 Bq m^−3^ in newly built dwellings (Radiation and Nuclear Safety Authority 2022). The third action level applied was 300 Bq m^−3^, based on the recommendation of European Union (Ruano-Ravina et al. 2017).

### Joint effect of residential radon and smoking

An analysis was performed to discern the impact of residential radon alone and the joint effect of residential radon and smoking on the number of avoidable radon-attributable lung cancers. The former was obtained by calculating the number of lung cancers due to residential radon assuming a similar background incidence for current and former smokers as among never-smokers. The difference between the estimates of all radon-attributable lung cancers and lung cancers due to radon alone was assumed to represent the number of lung cancers due to joint effect of radon and smoking. A similar relative risk coefficient implies that the joint effect (interaction) of radon and smoking is multiplicative.

In addition, the number of avoidable lung cancers assuming different ERR estimates for smokers and non-smokers was estimated. The estimates were calculated assuming that the corrected estimate (16% increase in lung cancer risk per 100 Bq m^−3^ increase in radon concentration) represented a weighted average of ERRs of the groups. Applying the estimates from pooled studies on miners (Radiation and on the E of A 2020), an ERR estimate for both ERR among smokers (10%) and non-smokers (17%) was derived. For the analysis, current smokers and former smokers were considered as a single group.

### Quantification of uncertainty in the number of radon-attributable lung cancers

The estimates of radon-attributable lung cancers held considerable uncertainty stemming from several sources. To quantify this uncertainty, a computational approach was used that makes minimal modelling assumptions and relies on the observed data.

The primary sources of uncertainty were the ERR estimates used to assess the relative lung cancer risk due to radon. Furthermore, the data on smoking prevalence included annual variation in addition to uncertainty in the actual estimates from the surveys and model-based imputations for missing data. These factors resulted in uncertainty around the smoking exposure estimates, which contribute fundamentally to the risk of lung cancer. Third, regional radon concentrations were based on two surveys with somewhat different results.

These sources of uncertainty were addressed by establishing, for each parameter, a distribution that reflects the uncertainty to be used in Monte Carlo simulations. At every Monte Carlo run, a set of independent parameter estimates were drawn from their distributions. Based on these estimates, the number of avoidable radon-attributable lung cancers were calculated as described above. Per simulation, the Monte Carlo procedure was repeated 10,000 times, yielding a distribution of radon-attributable lung cancers. From the resulting distribution, the mean and 2.5% and 97.5% quantiles of the distribution were reported. The latter two were taken to represent the 95% uncertainty interval of attributable lung cancers.

A total of five simulations (A, B, C, D and E) were run, and for each simulation, a different distribution of ERR was used to reflect the uncertainty. For the simulations A and B, these distributions were solely based on uncorrected and corrected ERRs and their confidence intervals from Darby et al., and were taken as normal distributions with means 8 and 16 and variances 3.3^2^ and 6.6^2^ (Darby et al. 2006). For the simulations C and D, distributions were obtained by pooling the uncorrected (C) and corrected estimates (D) from the European and North American pooled residential studies. The North American corrected estimate was based on a restricted sample of subjects living in 1–2 homes covered with α-track monitors for at least 20 years and was therefore less affected by bias (Krewski et al. 2006). From these, normal distributions with means 9.5 and 17 were assumed for lung cancers and small cancer carcinomas, respectively, and variances 2.43^2^ and 6.18^2^, respectively. For the simulation E (small cell carcinomas) the pooled estimate (a normal distribution with mean 27 and variance 13.7^2^) from European and North American studies was used. (Krewski et al. 2005; Darby et al. 2006).

In each Monte-Carlo run, the uncertainty in smoking exposure was accounted for by randomly sampling a year with equal probabilities between 2000 and 2015, and using the smoking prevalence from the sampled year as an estimate of smoking exposure. Thus, changes in smoking trends during the time interval were accounted for.

To address the uncertainty in radon exposure, dwelling type and region-specific distributions for the radon concentrations were established based on the 1991 and 2006 surveys, and sampled an estimate from these strictly bimodal distributions.

At extreme cases, some simulations yielded negative estimates. These estimates were truncated to zero, since there is no credible evidence that radon could protect from lung cancer*.* Uncertainty intervals were reported separately for the total number of all lung cancers and small cell carcinomas and for numbers of lung cancers in each dwelling type, sex and smoking-specific strata. All analyses were carried out using R statistical software (4.0.3).

## Results

### Characteristics of the study population

The majority of the Finnish population (63%) resided in houses in 2017 (Table 1) and over one-fifth was aged 65 years or older. Overall, approximately 12% of Finnish population was estimated to be current smokers in 2007. Smoking was more common among residents of flats (143 per 1000) than houses (99 per 1000).

**Table 1:** Population and number of lung cancers by demographic group in 2017, *n* (%)

	Population		Lung cancers		Small cell carcinomas	
	Flats	Houses	Flats	Houses	Flats	Houses
Age group						
0–44	1,081,561 (56)	1,712,004 (51)	17 (1)	13 (1)	2 (1)	1 (1)
45–54	199,610 (10)	473,103 (14)	54 (4)	39 (3)	16 (8)	12 (7)
55–64	224,522 (12)	482,351 (14)	265 (18)	206 (17)	39 (18)	30 (18)
65–74	231,664 (12)	422,480 (13)	622 (41)	489 (41)	97 (46)	75 (45)
75–84	140,202 (7)	194,252 (6)	416 (28)	334 (28)	52 (24)	41 (25)
85–	57,908 (3)	61,420 (2)	126 (8)	113 (9)	7 (3)	6 (4)
Sex						
Men	907,034 (47)	1,691,201 (51)	955 (64)	751 (63)	129 (61)	101 (61)
Women	1,028,433 (53)	1,654,409 (49)	546 (36)	442 (37)	83 (39)	65 (39)
Smoking						
Current	278,089 (14)	332,677 (10)	690 (46)	425 (36)	106 (50)	64 (39)
Ex	474,505 (25)	766,566 (23)	581 (39)	499 (42)	78 (37)	68 (41)
Never	1,182,873 (61)	2,246,367 (67)	230 (15)	268 (22)	28 (13)	33 (20)
Total	1,935,467	3,345,610	1501	1193	212	166

Smoking exposure grouping and the number of lung cancers among current smokers, former smokers and never smokers were estimated using smoking prevalence data from 2007. Due to rounding, sums in each category do not necessarily add to total and percentages to 100

A total of 2694 incident lung cancers were diagnosed in 2017, out of which nearly 80% occurred among persons 65 years or older (Table 1). The incidence of lung cancer was higher among men than women (66 vs. 37 per 100,000 population) and based on the estimations, higher among residents of flats than of houses (77 vs. 36) and notably more common among current smokers compared to former smokers and never smokers (183 vs. 87 vs. 15 per 100,000). Out of all lung cancers, 14% were small cell carcinomas and their distributions in all examined strata differed only slightly from that of all lung cancers.

### Residential radon exposure

**Fig. 2:**
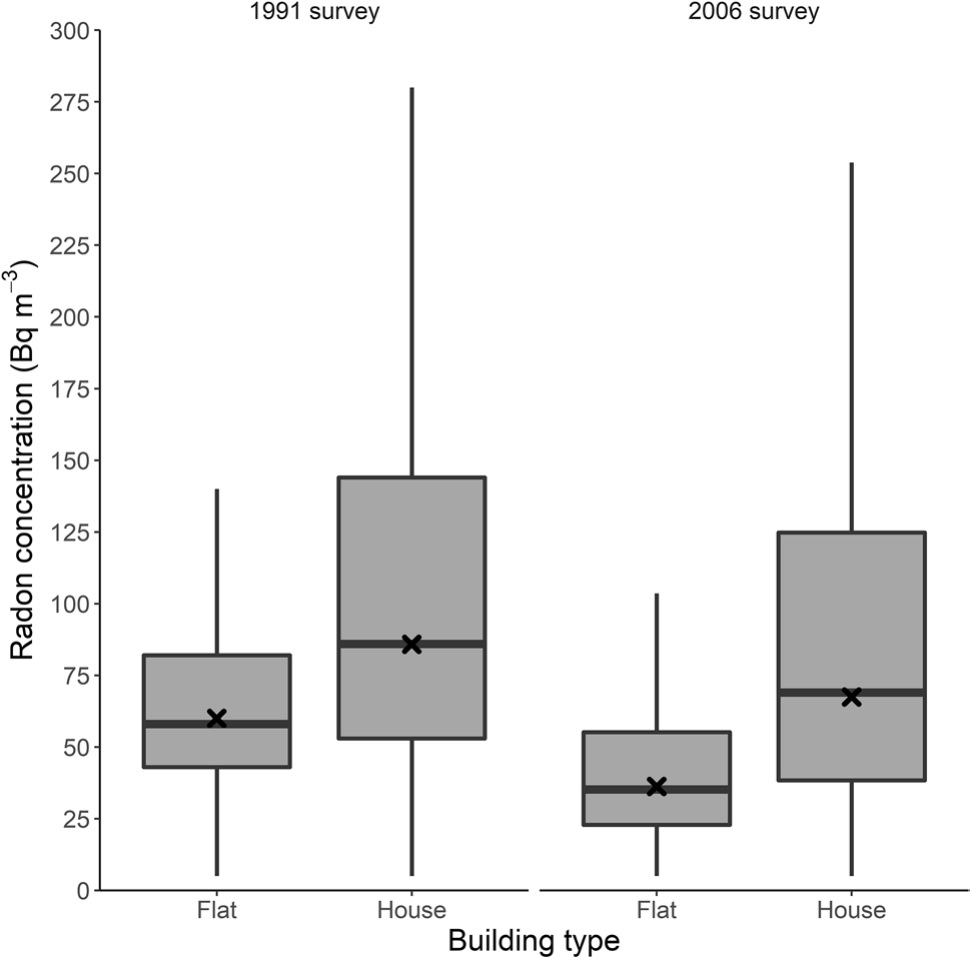
Radon concentrations by survey and dwelling type. X marks indicate geometric means

The geometric mean radon concentrations were approximately two-fold in houses compared to flats in both surveys (Fig. 2). In the 1991 survey, the geometric mean was 62 Bq m^−3^ for flats and 109 Bq m^−3^ for houses. In 2006, the means were lower overall—37 Bq m^−3^ and 78 Bq m^−3^—but their ratio remained similar. Variability in radon concentrations between houses was considerably larger compared to variability between flats.

**Fig. 3:**
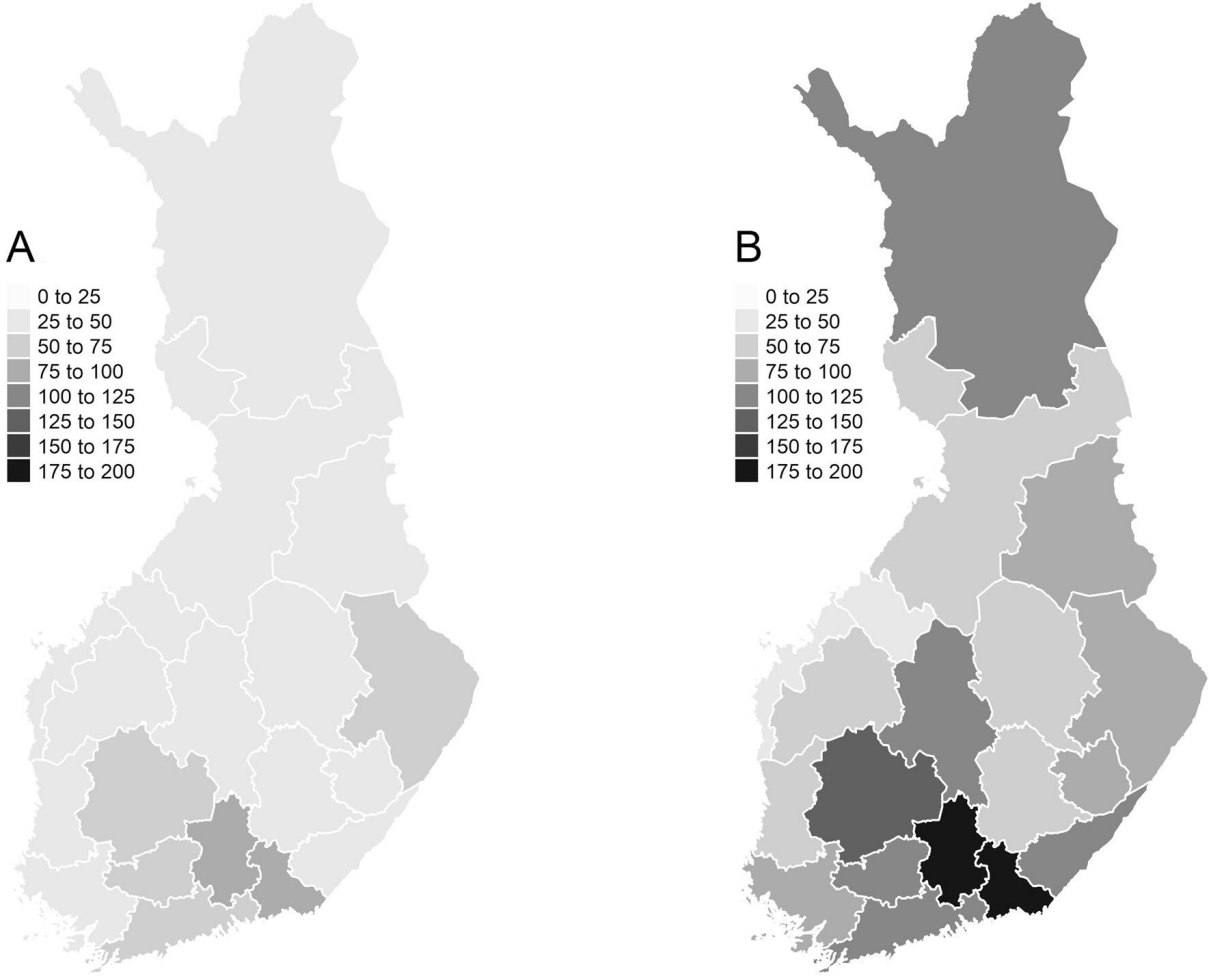
Geometric mean radon concentrations (Bq m^−3^) within flats (**A**) and houses (**B**) by region. Radon concentrations are averages of the geometric means of 1991 and 2006 surveys

Radon concentrations of both dwelling types varied substantially by region (Fig. 3). Geometric mean radon concentrations of both flats and houses were highest in southern parts of Finland (86 Bq m^−3^ for flats and 194 Bq m^−3^ houses) and lowest in Western Finland (32 and 44 Bq m^−3^). Difference between dwelling types was largest (2.7-fold) in Southern Finland. Regional variation in radon concentration was similar in both surveys (data not shown).

### Avoidable lung cancers attributable to residential radon

Assuming an ERR of 8.4% per 100 Bq m^−3^ and the average of radon concentrations from the two surveys, a total of 152 radon-attributable lung cancers was estimated in Finland in 2017, of which 97 could be avoided if radon exposure above 25 Bq m^−3^ was eliminated (Table 2). When assuming ERR of 16% per 100 Bq m^−3^, 170 out of 273 radon-attributable lung cancers could be avoided. The numbers of avoidable radon-attributable lung cancers correspond to population attributable fractions of 0.04 (ERR = 8.4%) and 0.06 (ERR = 16%) and correspond to 64% and 62% of all radon-attributable lung cancers (i.e. radon levels > 0) and 4% and 6% of all lung cancers.

**Table 2:** Estimated number of avoidable lung cancers and small cell carcinomas attributable to residential radon by demographic group

	Lung cancers, *n* (%)								
	1990 survey			2006 survey			Average of the surveys		
	Flats	Houses	Total	Flats	Houses	Total	Flats	Houses	Total
Overall	1501	1193	2694	1501	1193	2694	1501	1193	2694
Overall, small cell carcinomas	212	166	378	212	166	378	212	166	378
Radon-attributable									
ERR = 8.4%	74 (5)	102 (9)	176 (7)	45 (3)	83 (7)	128 (5)	60 (4)	93 (8)	152 (6)
ERR = 16%	134 (9)	180 (15)	314 (12)	83 (6)	148 (12)	231 (9)	109 (7)	164 (14)	273 (10)
Small cell carcinomas (ERR = 31%)	33 (15)	41 (25)	74 (20)	21 (10)	35 (21)	55 (15)	27 (13)	38 (23)	65 (17)
Radon-attributable at 25 Bq m^3^									
ERR = 8.4%	31 (2)	25 (2)	55 (2)	31 (2)	25 (2)	55 (2)	31 (2)	25 (2)	55 (2)
ERR = 16%	58 (4)	46 (4)	104 (4)	58 (4)	46 (4)	104 (4)	58 (4)	46 (4)	104 (4)
Small cell carcinomas (ERR = 31%)	15 (7)	12 (7)	26 (7)	15 (7)	12 (7)	26 (7)	15 (7)	12 (7)	26 (7)
Avoidable radon-attributable									
ERR = 8.4%	43 (3)	78 (7)	121 (4)	14 (1)	58 (5)	72 (3)	29 (2)	68 (6)	97 (4)
ERR = 16%	77 (5)	134 (11)	211 (8)	25 (2)	102 (9)	127 (5)	52 (3)	118 (10)	170 (6)
Small cell carcinomas (ERR = 31%)	18 (9)	30 (18)	48 (13)	6 (3)	23 (14)	29 (8)	12 (6)	26 (16)	39 (10)
Age group									
0–44	1 (1)	2 (1)	2 (1)	0 (1)	1 (1)	1 (1)	1 (1)	1 (1)	2 (1)
45–54	3 (4)	5 (3)	8 (4)	1 (4)	4 (3)	5 (4)	2 (4)	4 (3)	6 (4)
55–64	13 (18)	23 (17)	36 (17)	4 (17)	17 (17)	22 (17)	9 (17)	20 (17)	29 (17)
65–74	32 (42)	56 (42)	88 (42)	11 (43)	42 (42)	53 (42)	22 (42)	49 (42)	71 (42)
75–84	21 (27)	36 (27)	57 (27)	7 (26)	28 (27)	34 (27)	14 (27)	32 (27)	46 (27)
85–	7 (8)	13 (9)	19 (9)	2 (9)	10 (10)	12 (9)	4 (8)	11 (9)	16 (9)
Sex									
Men	48 (63)	82 (62)	131 (62)	16 (62)	63 (62)	79 (62)	32 (63)	73 (62)	105 (62)
Women	28 (37)	51 (38)	80 (38)	10 (38)	39 (38)	49 (38)	19 (37)	45 (38)	65 (38)
Smoking									
Current	35 (46)	48 (36)	83 (39)	12 (46)	36 (36)	48 (38)	24 (46)	42 (36)	66 (39)
Former	29 (38)	56 (42)	85 (40)	10 (39)	42 (42)	52 (41)	20 (38)	49 (42)	69 (41)
Never	12 (15)	30 (23)	42 (20)	4 (16)	23 (23)	27 (21)	8 (15)	27 (23)	35 (21)

The number of lung cancers by age, sex and smoking status were calculated using ERR of 16%. Due to rounding, sums in each category do not necessarily add to total and percentages to 100

Nearly 40% of avoidable radon-attributable lung cancers occurred among current smokers (12% of population), regardless of the ERR estimate applied (Table 2, Online Resource 1). The proportion of avoidable radon-attributable lung cancers among current smokers was over 10 percentage points higher among residents in flats than in houses. However, the proportions among both former smokers and never smokers were 4% points lower in flats than houses.

**Fig. 4:**
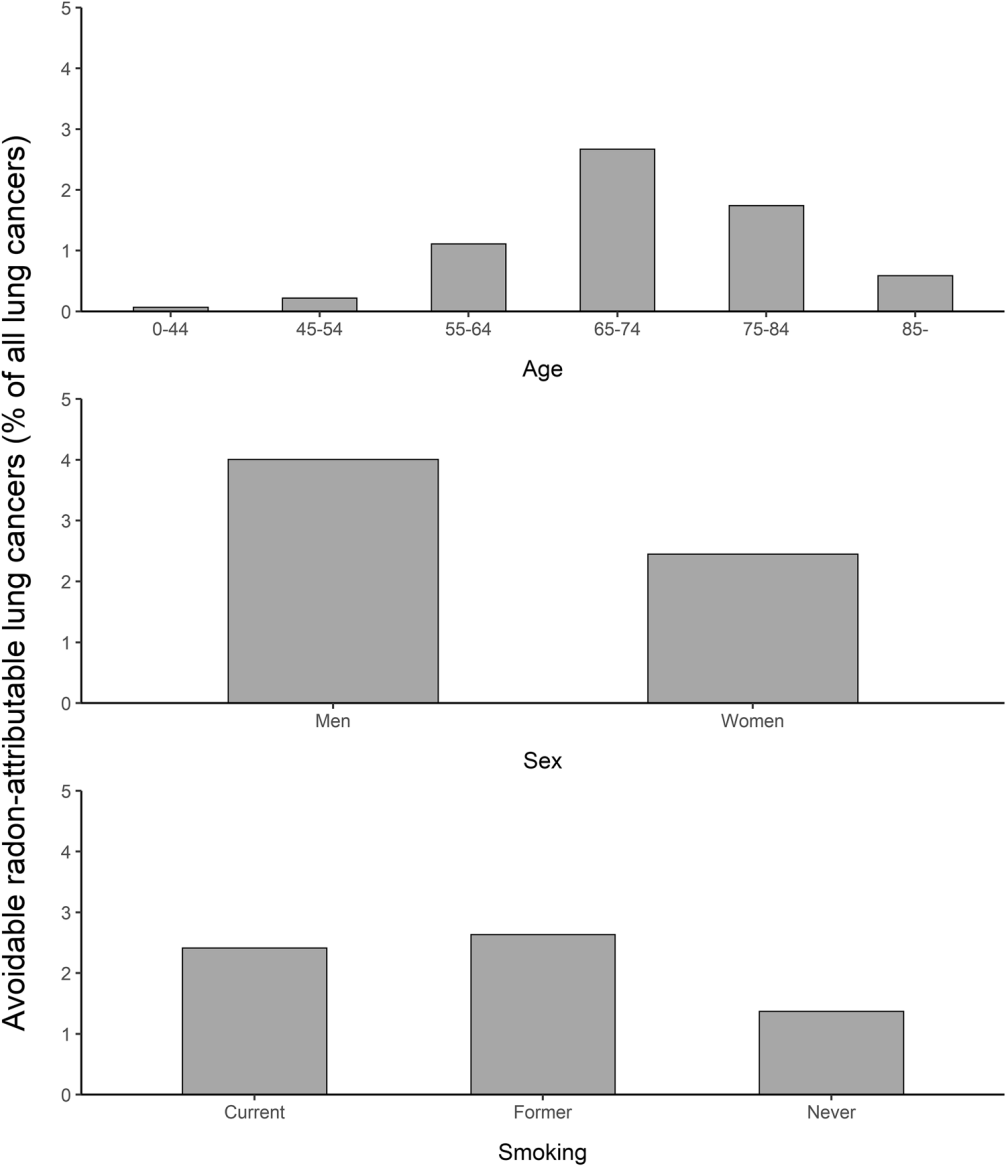
Proportion of avoidable radon-attributable lung cancers (calculated as a difference between radon-attributable lung cancers at observed radon levels and 25 Bq m^−3^) by age, sex and smoking status (ERR = 16%; taken from Darby et al. 2006). Calculations were based on averages of the geometric means of radon concentrations from 1991 to 2006 surveys

Regardless of the risk estimate employed, most of the avoidable radon-attributable lung cancers occurred among people living in houses (70%), among people at age 65 or older (78%) and among men (62%) (Fig. 4, Table 2). The proportions of radon-attributable lung cancers by age and sex differed only slightly between the dwelling types. The number of radon-attributable lung cancers was approximately 1.5-fold when using radon concentrations from 1991 survey compared to 2006 survey. Similar between-survey difference was observed with both ERR estimates.

A total of 65 small cell carcinomas were estimated to be attributable to radon in Finland in 2017 assuming average of surveys radon concentrations (ERR = 30%, PAF = 0.12) (Table 2, Online Resource 2), of which 39 could be avoided by reducing radon exposure to 25 Bq m^−3^. Most avoidable small cell carcinomas (67%) occurred among people dwelling in houses, those aged age 65 or older (74%) and men (59%). Small cell carcinomas were also clearly more common among current smokers. When calculated based on the 1991 survey, the number of avoidable small cell carcinomas was 1.5-fold compared to the 2006 survey.

### Impact of residential radon mitigation on the number of lung cancers

**Fig. 5:**
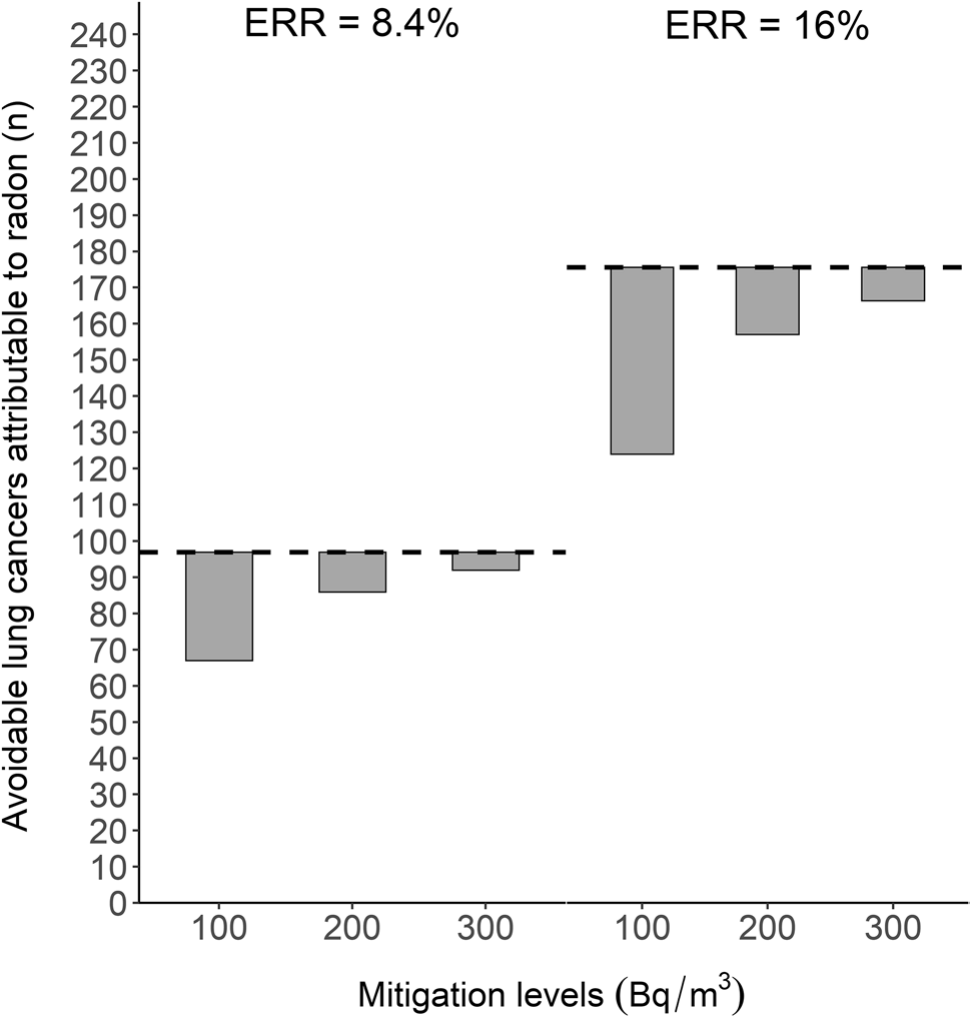
The potential decrease in the avoidable number of radon-attributable lung cancers (calculated as a difference between radon-attributable lung cancers at observed radon levels and 25 Bq m^−3^) if radon exposure levels were mitigated to levels 100, 200 and 300 Bq m^−3^ calculated with excess relative risks (ERR) of 8.4% (**A**) and 16% (**B**) per 100 Bq m^−3^ (taken from Darby et al. 2006) Horizontal dashed lines indicate avoidable radon-attributable lung cancers on observed radon concentration levels

With ERR 8.4% per 100 Bq m^−3^ and the average of radon concentrations from the two surveys, 5, 11 and 30 radon-attributable lung cancers could be prevented if the highest radon concentrations were mitigated to 300 Bq m^−3^, 200 Bq m^−3^ or 100 Bq m^−3^, respectively (Fig. 5). With ERR of 16%, 9, 18 and 50 radon-attributable lung cancers due to radon could be prevented with the same mitigation levels. With both relative risk estimates, these correspond to approximately 30% decrease in radon-attributable lung cancers in the most optimistic scenario.

### Joint effect of residential radon and smoking

**Fig. 6:**
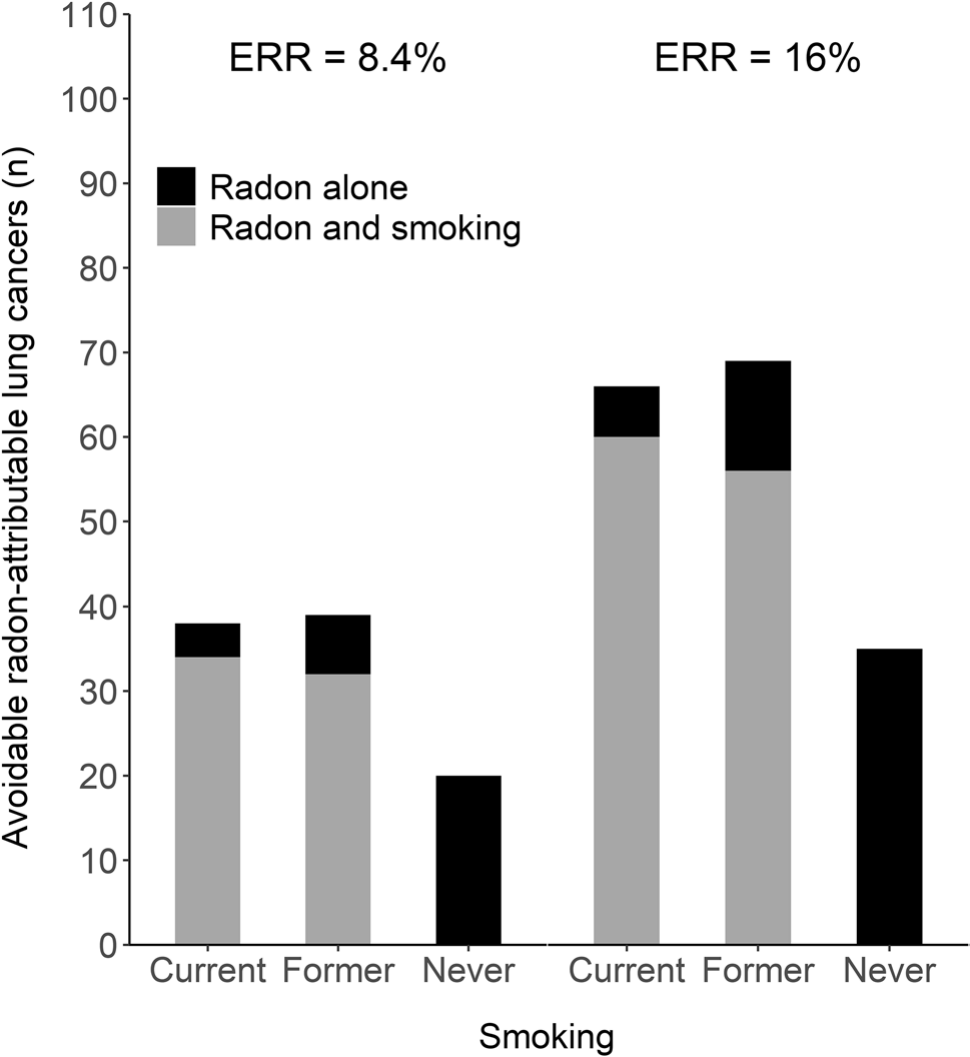
Avoidable lung cancers attributable to residential radon alone and due to joint effect of residential radon and smoking assuming excess relative risk (ERR) of 8.4% (**A**) and 16% (**B**) per 100 Bq m^−3^ increase in radon concentration (taken from Darby et al. 2006). Calculations were based on averages of the geometric means of radon concentrations of 1991 and 2006 surveys

Regardless of the radon risk estimate used, the proportion of avoidable radon-attributable lung cancers among current smokers due to radon alone was approximately 9% of the total lung cancers due to radon among them (Fig. 6). The remainder of the radon-induced lung cancer risk in smokers was attributed to the joint effect of radon and smoking, as it was deduced that it would have been avoided by eliminating either of the exposures. Among former smokers, roughly a fifth of the radon-induced cases could be attributed to radon alone and the rest to the interaction between radon and smoking. Incidence for avoidable lung cancers due to joint effect of radon and smoking was 12.4 and 5.6 per 100,000 population for current and former smokers (with 16% ERR and average of surveys radon concentrations), respectively.

When assuming separate ERR for smokers and never-smokers, the estimate of avoidable radon-attributable lung cancers was over 40 cases lower compared to the situation with no difference in ERRs between smoking groups (Table 3). In particular, the estimate changed in the smoker group (− 45 cases) while only slightly in the never-smoker group (+ 2 cases).

**Table 3:** Estimated number of avoidable lung cancers and small cell carcinomas attributable to residential radon by demographic group when assuming ERR of 10% for smokers and ERR 17% for non-smokers

	Lung cancers, *n* (%)								
	1990 survey			2006 survey			Average of the surveys		
	Flats	Houses	Total	Flats	Houses	Total	Flats	Houses	Total
Overall	1501	1193	2694	1501	1193	2694	1501	1193	2694
Radon-attributable	96 (6)	135 (11)	231 (9)	59 (4)	111 (9)	169 (6)	77 (5)	123 (10)	200 (7)
Avoidable radon-attributable	55 (4)	102 (9)	157 (6)	18 (1)	77 (6)	95 (4)	37 (2)	90 (8)	127 (5)
Age group									
0–44	1 (1)	1 (1)	2 (1)	0 (1)	1 (1)	1 (1)	0 (1)	1 (1)	1 (1)
45–54	2 (4)	3 (3)	5 (3)	1 (4)	3 (3)	3 (4)	1 (4)	3 (3)	4 (3)
55–64	9 (17)	17 (16)	26 (16)	3 (16)	13 (16)	16 (16)	6 (17)	15 (16)	21 (16)
65–74	22 (41)	41 (40)	63 (40)	8 (41)	31 (40)	38 (40)	15 (41)	36 (40)	51 (40)
75–84	15 (28)	29 (28)	44 (28)	5 (27)	22 (28)	27 (28)	10 (28)	25 (28)	36 (28)
85-	5 (10)	11 (11)	16 (10)	2 (10)	8 (11)	10 (11)	4 (10)	10 (11)	13 (11)
Sex									
Men	34 (61)	61 (60)	94 (60)	11 (60)	46 (60)	57 (60)	22 (61)	53 (60)	76 (60)
Women	22 (39)	41 (40)	63 (40)	7 (40)	31 (40)	38 (40)	15 (39)	36 (40)	51 (40)
Smoking									
Smokers	43 (77)	70 (69)	113 (72)	14 (77)	53 (68)	67 (70)	29 (77)	61 (69)	90 (71)
Never-smokers	12 (23)	32 (31)	44 (28)	4 (23)	24 (32)	29 (30)	8 (23)	28 (31)	37 (29)

Smokers group comprises both current smokers and former smokers. Due to rounding, sums in each category do not necessarily add to total and percentages to 100

### Quantification of uncertainty in the number of radon-attributable lung cancers

With each estimate for radon risk, the simulations gave wide uncertainty intervals, reflecting the substantial uncertainty in the estimates of radon-attributable lung cancers (Table 4). While the mass of the distribution ranges from few tens to approximately two hundred, the highest plausible overall value is as high as 262. The mean number of avoidable radon-attributable cases obtained from simulations A, B and D were slightly lower (93, 162, 33, respectively) compared to the estimates from the main analysis based on relative risk estimates 8.4%, 16% and 30% (97, 170, 39). The relative risk estimate for small cell carcinomas was too inaccurate to rule out the possibility of no lung cancer cases attributable to radon. The Simulation D that used pooled estimate from European and North American corrected estimates resulted in slightly higher and more precise estimate for the number of avoidable lung cancers compared to Simulation B.

**Table 4:** Simulated point estimates and uncertainty intervals (*M* = 10,000) for the number of radon-attributable lung cancers in Finland in 2017 by demographic group

	Avoidable radon-attributable lung cancers, mean (95% uncertainty interval)				
	Simulation A	Simulation B	Simulation C	Simulation D	Simulation E
Overall	93 (23, 155)	162 (35, 261)	105 (55, 150)	170 (56, 262)	33 (0, 55)
Dwelling					
Houses	67 (16, 110)	114 (25, 183)	75 (40, 107)	121 (40, 183)	23 (0, 37)
Flats	26 (6, 45)	47 (10, 79)	30 (15, 43)	50 (16, 79)	10 (0, 17)
Sex					
Men	58 (14, 96)	100 (22, 162)	65 (34, 93)	106 (34, 163)	20 (0, 33)
Women	36 (9, 59)	61 (13, 99)	40 (21, 57)	65 (21, 100)	13 (0, 22)
Smoking					
Smokers	33 (8, 56)	57 (13, 95)	37 (19, 55)	60 (20, 96)	13 (0, 22)
Former	40 (9, 67)	69 (15, 113)	45 (23, 65)	73 (24, 113)	14 (0, 23)
Never	20 (5, 34)	35 (8, 58)	23 (12, 33)	37 (12, 58)	6 (0, 11)

Simulations were based on established normal distributions for the risk coefficients (A, B, C, D and E). In each simulation, (1) a year between 2000 and 2015 was randomly sampled with equal probabilities and smoking prevalence from sampled year was used and (2) radon concentrations were sampled from established region and dwelling type -specific bimodal distributions based on the 1991 and 2006 surveys. Negative estimates were truncated to zero. (A) Simulation based on the distribution of relative risk [8.4, 3.3^2^] for uncorrected radon exposure in the European pooled study. (B) Simulation based on the distribution of relative risk [16, 6.6^2^] for the radon exposure corrected for measurement error in the European pooled study. (C) Simulation based on the distribution of relative risk [9.5, 2.3^2^] pooled from European and North American studies. (D) Simulation based on the distribution of corrected relative risk estimates [17, 6.1^2^] pooled from the European and North American studies. (E) Simulation based on the distribution of relative risk [27, 13.7^2^] pooled from the European and North American studies (small cell carcinomas)

## Discussion

The results indicate that approximately 72–211 lung cancers or 3–8% of all cases were attributable to avoidable indoor radon exposure in Finland in 2017. These estimates are well below those published for Finland, e.g., in an international comparison (Gaskin et al. 2018). Despite the estimates deviate from those of Gaskin et al. mainly due to methodological differences, they provide a direct point of comparison on a national level.

Radon-attributable lung cancers mainly occurred among current and former smokers, among people at age 65 or older, and were more frequent in men than women, and among those living in houses than flats. Of small cell carcinomas, approximately 10% could be attributed to residential radon. These estimates are subject to uncertainty arising from several sources, most importantly uncertainty in radon exposure levels of the population, and magnitude of risk per unit exposure. An uncertainty interval accounting for all identifiable sources of error was estimated, and it indicated a considerable range of plausible values.

Despite relatively high national average radon levels in Finland, the estimates are comparable to previous studies conducted in France (Catelinois et al. 2006), Germany (Menzler et al. 2008), Switzerland (Menzler et al. 2008) and Korea (Kim and Ha 2018), but lower than most estimates published for Canada. The results are also well below those estimated for Finland in a global study (22–27% of all lung cancer deaths) (Gaskin et al. 2018). The differences are due to the methods including different risk models employed (derived from residential studies versus miner studies), choice of comparison levels (zero versus 25 Bq m^−3^) and incorporation of the inverse correlation between smoking and radon exposure, i.e. higher radon levels and lower smoking prevalence among residents of houses than flats, which has been ignored in most previous studies.

Many of the previous assessments could have overestimated the numbers of lung cancers attributable to residential radon. One of the issues that needs to be carefully incorporated in such assessments is the strongly skewed joint distribution of smoking prevalence and radon levels. In the study, 10% of Finns residing in houses were current smokers, while the prevalence for people residing in flats was 14%. Ignoring the inverse correlation would have resulted in a somewhat larger estimate of radon-attributable lung cancers (198 vs. 170 avoidable radon-attributable lung cancers when assuming ERR of 16% and average radon concentrations from 1991 to 2006 surveys). Socioeconomic factors probably explain the difference, as residents of houses are more likely to have higher education and income levels. This phenomenon is likely to be present also in other countries, and studies ignoring it may have overestimated the attributable fraction of lung cancers, while careful incorporation of this fact is a key strength of this study.

Another methodological issue is that in this study a reference level of 25 Bq m^−3^ was used, i.e. the numbers of radon-attributable lung cancers were estimated relative to this exposure level, chosen to represent the lowest attainable residential exposure levels. This means that the results should be interpreted as representing the number of lung cancers that could in principle be averted if residential radon levels were reduced to 25 Bq m^−3^ (given the other assumptions). Approximately 64% (ERR = 8.4%) and 62% (ERR = 16%) of the radon-attributable lung cancers were estimated to be avoidable this way. Several other studies have estimated the numbers compared with zero exposure, which gives higher, but less realistic estimates, as radon is not absent even from outdoor air. Hence, a complete elimination of radon exposure is unattainable even in theory.

The approach in this study was based on allocating the observed numbers of lung cancer cases by age, sex, region according to the distribution of risk determinants and their effect sizes, rather than applying risk coefficients to a baseline rate. This was chosen to constrain the values to a realistic range for each population subgroup. This approach effectively divides lung cancers into three groups, those attributable to smoking, radon, or other factors. Importantly, frequencies of cases estimated as radon-attributable are not dependent on those attributed to smoking.

Of the avoidable radon-attributable cases among current smokers, large majority was assigned to the joint effect of radon and smoking, while only approximately 1/10 could be attributed to radon alone. The latter were estimated assuming that lung cancer incidence among smokers would be similar to that among non-smokers. Among former smokers, approximately 20% of avoidable radon-attributable lung cancers was due to radon alone.

Of the radon-attributable lung cancers, a substantial proportion would be preventable by lowering the highest residential radon levels to the current or previous guideline values (100–300 Bq m^−3^). This has clear implications for radon policy: a strict enforcement of the reference levels to lower the highest exposures will have notable impact on the population-attributable risk. However, a more comprehensive policy aiming at minimizing radon exposure throughout the housing stock would be more effective (as low as reasonable achievable ALARA) (Gray et al. 2009; Pollard and Fenton 2014; Svensson et al. 2018).

This analysis is based on two large national population-based radon surveys conducted in 1990 and 2006 (Arvela et al. 1993; Kinnunen et al. 2009). Both surveys employed standardised methodology (though not identical in the two surveys) and correction for selection effects due to incomplete participation. The differences in estimated radon levels between the two surveys were higher than would be expected by chance alone. Possible explanations include differences in calibration method and temperature during the measurement periods (warmer temperatures are generally associated with lower radon levels). Newer buildings tend to have lower radon levels, but housing constructed between the surveys cannot explain the difference. Further uncertainty in application of the exposure estimates from population surveys include occupancy and exposure level outside home (ignored here).

For the effect of radon on lung cancer risk, the results of the European pooled analysis were applied, which was regarded as the best available estimate as it reflects residential radon exposure and is the largest published study on the topic. The Finnish residential radon levels are also comparable to the European study. Previous studies estimating population-attributable risk have mostly used BEIR VI risk models based on cohort studies of occupationally exposed miners with very high radon exposure levels, with subjects limited to men at working ages with very high smoking rates and lung cancer mortality rather than incidence as the endpoint (Beir 1999). The BEIR VI model gives higher (up to twice as large) risk coefficients per unit exposure than the European pooled analysis (or other residential studies); however, recent Pooled Uranium Miner Analysis reported very similar risk parameters to those of BEIR VI among modern miners exposed to more moderate radon exposures. (Richardson et al. 2021). It also incorporates parameters for time since exposure and duration of exposure that are not equally relevant or easily applicable for residential exposure. Applying the BEIR VI model requires assumptions about past levels of exposure to account for time since exposure, i.e., effects of past exposure, which is an additional source of uncertainty.

A linear dose–response is well supported by residential studies, with exposure levels generally below 1,000 Bq m^−3^. An inverse dose rate effect has been shown in miner studies (with a higher risk per unit exposure at lower activity concentrations), but it is not important for residential exposure levels. As for latency, miner studies have shown that the effect of radon is well represented by exposure 5–25 years earlier. In this study, average levels assumed to account for exposure during that period were used, with three alternative estimates based on the two national surveys and the average of the two (which can also be taken to represent exposure levels from the former 1990 survey until the time of the later survey, i.e., both as valid measures for the two time periods).

Similar to the European pooled study, both relative risk estimates corrected for measurement uncertainty (‘usual radon’) and those for observed radon (Darby et al. 2005) were applied. Good arguments for both approaches can be presented: observed, uncorrected estimates may be conservative, involve less assumptions and fit the Finnish radon measurements. On the other hand, radon measurements as indicators of past exposure are known to involve uncertainties due to e.g. temporal variation and correction for random error is used to reduce such uncertainty. Also, the corrected relative risk estimate is similar to a pooled estimate obtained in a meta-analysis (Gogna et al. 2019). Both results are shown to demonstrate the potential impact of measurement error, and both are well within the uncertainty margin estimated in the study. The approach in this study to estimate the uncertainty range was based on uncertainty in each of the components used to derive the population attributable risk (PAR) estimates, including radon level, relative risk per unit exposure and smoking prevalence (Krewski et al. 1999; Brand et al. 2005; Catelinois et al. 2006).

In the analysis, no interaction (effect modification) was assumed between radon and other risk factors, i.e. the radon-induced risk per unit exposure (on relative scale) was assumed constant for men and women, and across different age groups (age attained or at exposure) and independent of smoking status. This is consistent with studies of residential radon.

This study has several strengths. Differences between houses and flats were incorporated in both radon levels and smoking patterns, accounting for the inverse correlation between the two risk factors, and also used area-specific estimates of radon exposure and smoking prevalence (by dwelling type). Such fine granularity on both radon exposure and smoking level has not been achieved in earlier studies. Current and former smokers were also treated separately, with different baseline lung cancer risk incorporated, and temporal changes in smoking were considered. The lung cancer incidence data were obtained from a comprehensive, high-quality cancer registry (Leinonen et al. 2017) and enables more realistic estimation of the radon burden compared to studies relying on mortality data instead. Furthermore, uncertainty in the estimates of key parameters was quantified, including sampling variability and assumptions made, to provide an uncertainty interval. In previous studies, Catelinois et al (2006) considered the influence of uncertainties in both radon risk coefficients and radon concentration on the estimates, whereas Menzer et al. (2008) made a more stringent assumption that the only uncertainties were due to statistical uncertainties in the parameter estimates of ERR (Catelinois et al. 2006; Menzler et al. 2008). The computational approach for uncertainty intervals utilises both sources of variation but extends the approach by incorporation of uncertainty not only due to estimated smoking prevalence, but also the possible misspecification of the period for lagged effects of radon and smoking exposure.

A key issue in analysis of lung cancers attributable to radon is whether similar relative risk estimates are applicable for smokers and non-smokers. In miner studies, the excess relative risk estimates have been clearly larger for non-smokers than smokers. Several analyses among miners have suggested a sub-multiplicative interaction between smoking and radon (Schubauer-Berigan et al. 2009; Leuraud et al. 2011). In this study, risk estimates from the pooled analysis of European residential studies were applied, which showed no difference in relative risk between smokers and non-smokers. If the interaction is indeed sub-multiplicative also in residential setting, the approach would overestimate the risk in smokers and underestimate it for non-smokers. This would likely yield somewhat lower estimates of radon-attributable lung cancers due to the substantially higher baseline risk among smokers.

In the analysis, lung cancer incidence data in a single year was used for simplicity. The lung cancer incidence in Finland in 2017 was 27 per 100,000 for men and 14 per 100,000 in women (standardised to the world population) (Finnish Cancer Registry). These are slightly below those for countries with a very high human development index in general (Sung et al. 2021). The incidence among men is declining (roughly 2% per year on average from 1990), but increasing among women (approximately 5% annually) (Finnish Cancer Registry).

## Conclusion

In this study, radon-attributable lung cancer risk in Finland was estimated based on extensive and detailed data. However, the precision of the estimates was limited by uncertainties in estimates of exposure, relative risk and smoking. The estimates were smaller than earlier ones, which is mainly due to the lower radon levels and higher smoking prevalence in flats than houses, a difference which has been ignored in previous studies. In addition, a more realistic minimum level of 25 Bq m^−3^ instead of zero exposure was used as the point of comparison. Residential radon was estimated to account for 3–8% of all lung cancers in Finland with average indoor concentrations close to 50 Bq m^−3^ in flats and 90 Bq m^−3^ in houses. Most of the radon-attributable cases occur in current and former smokers, men, older ages and residents of houses. Among smokers, majority of the radon-related cases were attributable to the joint effect of radon and smoking. A substantial number (30%) of radon-attributable cases could be eliminated by decreasing radon exposure to 100 Bq m^−3^ action level. Validity of the estimates was enhanced by incorporation of detailed exposure data by region and consideration of the inverse correlation between radon levels and smoking prevalence.

We believe our methods allow obtaining realistic estimates of radon-attributable cancers and uncertainty range based on clearly defined parameters and distributions of their estimates. The results could be used to guide decision-making in prevention of lung cancers and risks associated to radon.

## Supplementary Information

Below is the link to the electronic supplementary material.

Supplementary file1 (DOCX 18 KB)

Supplementary file2 (DOCX 18 KB)
