# Supplementary material for: Lung cancer incidence attributable to residential radon exposure in Finland
Source: Radiat Environ Biophys. 2022 Nov 8;62(1):35–49. doi: 10.1007/s00411-022-01004-1 (PMC9950193; doi:10.1007/s00411-022-01004-1)
Supplement: Supplementary file 1 — Supplementary file1 (DOCX 18 KB) [file 411_2022_1004_MOESM1_ESM.docx]

***Online Resource 1.*** *The* *estimated* *number of* *avoidable* *lung cancer cases attributable to residential radon by demographic groups (excess relative risk = 8.4% per 100 Bq m^-3^) in 2017, n (%).*

|  | Lung cancers, n (%) | | | | | | | | |
| --- | --- | --- | --- | --- | --- | --- | --- | --- | --- |
|  | 1990 survey |  |  | 2006 survey |  |  | Average of the surveys | | |
|  | Flats | Houses | Total | Flats | Houses | Total | Flats | Houses | Total |
| Overall | 1501 | 1193 | 2694 | 1501 | 1193 | 2694 | 1501 | 1193 | 2694 |
| Radon-attributable | 74 | 102 | 176 | 45 | 83 | 128 | 60 | 93 | 152 |
| Radon-attributable at 25 Bq m^3 | 31 | 25 | 55 | 31 | 25 | 55 | 31 | 25 | 55 |
| Avoidable radon-attributable | 43 | 78 | 121 | 14 | 58 | 72 | 29 | 68 | 97 |
| Age group |  |  |  |  |  |  |  |  |  |
| 0-44 | 1 | 1 | 1 | 0 | 1 | 1 | 0 | 1 | 1 |
| 45-54 | 2 | 3 | 4 | 1 | 2 | 3 | 1 | 2 | 4 |
| 55-64 | 8 | 13 | 21 | 2 | 10 | 12 | 5 | 12 | 17 |
| 65-74 | 18 | 32 | 50 | 6 | 24 | 30 | 12 | 28 | 40 |
| 75-84 | 12 | 21 | 33 | 4 | 16 | 20 | 8 | 19 | 26 |
| 85- | 4 | 7 | 11 | 1 | 6 | 7 | 2 | 6 | 9 |
| Sex |  |  |  |  |  |  |  |  |  |
| Men | 27 | 48 | 75 | 9 | 36 | 45 | 18 | 42 | 60 |
| Women | 16 | 30 | 46 | 5 | 22 | 28 | 11 | 26 | 37 |
| Smoking |  |  |  |  |  |  |  |  |  |
| Current | 20 | 28 | 48 | 6 | 21 | 27 | 13 | 24 | 38 |
| Former | 17 | 32 | 49 | 5 | 24 | 30 | 11 | 28 | 39 |
| Never | 7 | 18 | 24 | 2 | 13 | 16 | 4 | 16 | 20 |

Number of radon-attributable lung cancers among current smokers, former smokers and never smokers were estimated using smoking

prevalence data from 2007. Avoidable radon-attributable lung cancers were calculated as difference between number of lung cancers at observed radon

concentrations and at concentration of 25 Bq m^-3^. Due to rounding, counts and percentages do not necessarily sum up to total and 100.
