# Supplementary material for: Lung cancer incidence attributable to residential radon exposure in Finland
Source: Radiat Environ Biophys. 2022 Nov 8;62(1):35–49. doi: 10.1007/s00411-022-01004-1 (PMC9950193; doi:10.1007/s00411-022-01004-1)
Supplement: Supplementary file 2 — Supplementary file2 (DOCX 18 KB) [file 411_2022_1004_MOESM2_ESM.docx]

***Online Resource 2.*** *The estimated number of avoidable small cell carcinomas attributable to residential radon by demographic group based on risk coefficient calculated for radon (excess relative risk = 31% per 100 Bq m^-3^) in 2017.*

|  | Small cell carcinomas | | | | | | | | |
| --- | --- | --- | --- | --- | --- | --- | --- | --- | --- |
|  | 1990 survey |  |  | 2006 survey |  |  | Average of surveys | | |
|  | Flats | Houses | Total | Flats | Houses | Total | Flats | Houses | Total |
| Overall | 212 | 166 | 378 | 212 | 166 | 378 | 212 | 166 | 378 |
| Radon-attributable | 33 | 41 | 74 | 21 | 35 | 55 | 27 | 38 | 65 |
| Radon-attributable at 25 Bq m^3 | 15 | 12 | 26 | 15 | 12 | 26 | 15 | 12 | 26 |
| Avoidable radon-attributable | 18 | 30 | 48 | 6 | 23 | 29 | 12 | 26 | 39 |
| Age group |  |  |  |  |  |  |  |  |  |
| 0-44 | 0 | 0 | 0 | 0 | 0 | 0 | 0 | 0 | 0 |
| 45-54 | 1 | 2 | 3 | 0 | 2 | 2 | 1 | 2 | 3 |
| 55-64 | 3 | 5 | 9 | 1 | 4 | 5 | 2 | 5 | 7 |
| 65-74 | 8 | 13 | 22 | 3 | 10 | 13 | 6 | 12 | 18 |
| 75-84 | 4 | 7 | 12 | 1 | 6 | 7 | 3 | 7 | 10 |
| 85- | 1 | 1 | 2 | 0 | 1 | 1 | 0 | 1 | 1 |
| Sex |  |  |  |  |  |  |  |  |  |
| Men | 11 | 18 | 28 | 4 | 14 | 17 | 7 | 16 | 23 |
| Women | 7 | 12 | 19 | 2 | 9 | 12 | 5 | 11 | 16 |
| Smoking |  |  |  |  |  |  |  |  |  |
| Current | 9 | 11 | 20 | 3 | 9 | 12 | 6 | 10 | 16 |
| Former | 7 | 12 | 19 | 2 | 9 | 12 | 5 | 11 | 15 |
| Never | 2 | 6 | 8 | 1 | 5 | 6 | 2 | 5 | 7 |

Number of radon-attributable small cell carcinomas among current smokers, former smokers and never smokers were estimated using

smoking prevalence data from 2007. Avoidable radon-attributable lung cancers were calculated as difference between number of lung cancers at

observed radon concentrations and at concentration of 25 Bq m^-3^. Due to rounding, counts and percentages do not necessarily sum up to total and 100.
